# Psychosocial Factors in End-Stage Kidney Disease Patients at a Tertiary Hospital in Australia

**DOI:** 10.1155/2016/2051586

**Published:** 2016-10-11

**Authors:** Charan Bale, Alexandra Douglas, Dev Jegatheesan, Linh Pham, Sonny Huynh, Atul Mulay, Dwarakanathan Ranganathan

**Affiliations:** ^1^Royal Brisbane and Women's Hospital, Herston, Brisbane, QLD, Australia; ^2^Dr. D. Y. Patil Medical College, Pune, India

## Abstract

*Aim*. This study seeks to review the psychosocial factors affecting patients with end-stage kidney disease (ESKD) from a tertiary hospital in Australia.* Methods*. We audited patients with ESKD, referred to social work services from January 2012 to December 2014. All patients underwent psychosocial assessments by one, full-time renal social worker. Patient demographics, cumulative social issues, and subsequent interventions were recorded directly into a database.* Results*. Of the 244 patients referred, the majority were >60 years (58.6%), male (60.7%), born in Australia (62.3%), on haemodialysis (51.6%), and reliant on government financial assistance (88%). Adjustment issues (41%), financial concerns (38.5%), domestic assistance (35.2%), and treatment nonadherence (21.3%) were the predominant reasons for social work consultation. Younger age, referral prior to start of dialysis, and unemployment were significant independent predictors of increased risk of adjustment issues (*p* = 0.004, <0.001, and =0.018, resp.). Independent risk factors for treatment nonadherence included age and financial and employment status (*p* = 0.041, 0.052, and 0.008, resp.).* Conclusion*. Psychosocial and demographic factors were associated with treatment nonadherence and adjustment difficulties. Additional social work support and counselling, in addition to financial assistance from government and nongovernment agencies, may help to improve adjustment to the diagnosis and treatment plans as patients approach ESKD.

## 1. Introduction

Patients with end-stage kidney disease (ESKD) are exposed to multiple physical and psychological stressors as a result of their illness [1]. Treatment of ESKD in the form of dialysis imposes considerable stress, including potential changes in family relations, social interactions, and occupational demands [1]. The “biopsychosocial” impact of ESKD has been proposed to account for its poorer quality of life (QoL) compared to patients with other chronic diseases [1, 2]. Furthermore, survey data has shown significant correlation between poorer QoL and higher morbidity and mortality in ESKD [3].

As opposed to the mostly invariant biological risk factors, modifiable psychosocial factors may provide avenues for successful intervention and improved clinical outcomes in this population [4].

The renal social worker is the patients' advocate, serving as a bridge in communicating individual's needs to the medical and allied health team [5]. The social worker's expertise encompasses instrumental, informational, and emotional support [6]. Naik et al. commented that “a multidisciplinary team approach is critical to the overall care and QoL of patients with ESKD. Social workers play a central role in the care of these patients, which may be further enhanced by engaging them in the measurement and monitoring of QoL” [7].

There is a paucity of data pertaining to the psychosocial factors affecting patients with ESKD in Australia. This study seeks to identify these issues using the renal social work database of a tertiary hospital. We identify various reasons for initial referral, subsequent consultations, and interventions performed by the renal social worker over a three-year period. We then compare differences across patient demographics and modalities of ESKD management. Ultimately, the study would further explore the significance of social work involvement in the care of patients with ESKD and lead to future improvements in service delivery.

## 2. Methods

We conducted a single centre retrospective audit of the patients with ESKD (chronic kidney disease stage-V (CKD-V)) who were referred to one, full-time renal social worker from January 2012 to December 2014 at Royal Brisbane & Women's Hospital, Queensland, Australia. Ethics approval was obtained from the Human Research and Ethics committee.

Referrals for social work input were made either by healthcare staff (medical and allied health) or directly by patients and/or family members. The reason for referral varied and many individuals were referred for multiple reasons over the study period. All patients underwent an initial psychosocial assessment by the social worker, typically a 60-minute consultation where issues at index were identified. Subsequent referrals and clinical encounters were recorded directly into a database as they arose. Each issue was analysed separately in the case where an individual patient had many issues identified over the study period.

Social work interventions included instrumental, informational, and emotional supports. Instrumental support included patient advocacy; assistance with paper work/forms; referrals to relevant government agencies (e.g., Department of Housing, welfare services); and allied health services (e.g., psychologist, dietician, and occupational therapist). Informational support included provision of helpful resources across various domains (e.g., predialysis education, treatment adherence, aged care services, management of finances, and employment prospects). Emotional support was provided through counselling sessions and organising family meetings (e.g., for those with adjustment issues, caregiver stress, and palliative care discussions). All clinical reviews, documentation, and data collation were performed by the same social worker over the study period. Deidentified data was transposed into a spreadsheet, including patient demographics, social history, ESKD management modality, summary of social worker encounters, and the respective interventions carried out.

This study only included participants with ESKD defined by estimated glomerular filtration rate (eGFR) < 15 mL/min/1.73 m^2^ or those with a functioning renal transplant (CKD-Vt). Patients were subclassified into predialysis, maintenance haemodialysis (HD), peritoneal dialysis (PD), renal transplant, or palliative.

Adherence is defined as “the extent to which a patient complies with the prescribed treatment under limited supervision” [8]. Limited supervision involves monitoring a patient in the community, for example, review at an outpatient appointment or during outpatient dialysis. Adherence also can be defined “as the extent to which a person's behaviour (taking medication, following a diet, and/or executing lifestyle changes) corresponds with agreed recommendations from a healthcare provider” [9]. Adjustment is described in relation to how the patient adapts to the multitude of stressors posed by the routine and restrictions of treatment [10]. Referrals for treatment nonadherence and adjustment were made at the discretion of the treating team, typically when this had serious consequences on the patient's health outcome, for example, hospitalisation due to nonadherence or difficulty coping with life changes associated with ESKD.

Domestic assistance is a service aimed at helping people to remain independent in their home, by helping with the essential light house work tasks necessary to maintain hygiene and safety standards in the home [11].

### 2.1. Statistical Analysis

Baseline variables are described as proportions or mean (SD) as appropriate. We used cross tabs with chi-square test to assess association of demographic variables with socioeconomic issues that were identified. We used univariate and multivariate logistic regression to determine predictors of adjustment issues and nonadherence. Age, gender, country of origin, financial status, employment status, reimbursement plan, referral before or after starting renal replacement therapy (RRT), and marital status of the patients were considered for inclusion in multivariate model. Since financial status and employment status were highly correlated, separate multivariate models were constructed for each of them to avoid colinearity. A *p* value < 0.05 was considered as statistically significant and odds ratios with 95% confidence interval were calculated.

## 3. Results

The study included 244 patients (148 men) with mean age 62.4 (16.9) years. The majority of them were Australian by birth (152). The majority (61.6%) of referrals to social worker were made after dialysis commencement or transplantation. Baseline characteristics of the patients are shown in Table 1.  Table 2 shows issues identified after evaluation of the patients by the social worker.

**Figure 1:**
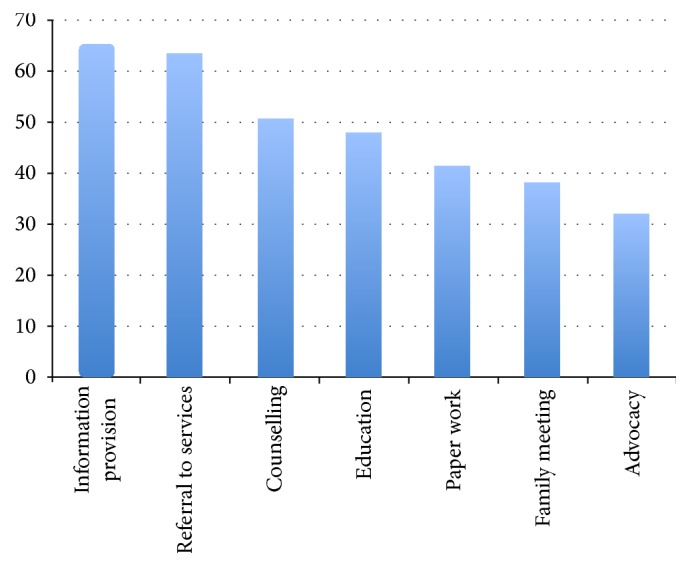
Breakdown of social work support services as a percentage. All patients received initial psychosocial assessment (not included in graphic).

Need for transportation assistance was most prevalent for those patients on HD (46/126 [36.5%]), followed by PD (15/60 [25%]) and transplant patients (7/32 [21.8%]), a significant difference between modality of RRT (*p* = 0.015) and a more commonly identified issue in patients referred prior to starting RRT (*p* = 0.004). Child protection was needed significantly more often if the country of birth was other than Australia (5/92 [5.4%] versus 1/152 [0.66%]; *p* = 0.03). The breakdown of social work interventions is listed in Figure 1.

Adjustment issues problems were the commonest problem identified in 41% of patients. Patients referred prior to starting RRT were more likely to have adjustment problems than those referred after commencement of RRT (72.3% versus 21.3%; *p* < 0.001, univariate analysis). In multivariate logistic regression, age, referral prior to commencement of RRT, and financial and employment status independently predicted the odds of having adjustment issues (Tables 3(a) and 3(b)). Separate models were created for financial status and employment status to avoid colinearity since the two were highly correlated. Increasing age was associated with a significantly decreased risk of having adjustment issues. Compared to aged pension, patients with financial stability (salary or savings) were significantly less likely to have adjustment issues. Compared to employed patients, unemployed patients were significantly more likely to have adjustment issues (odds ratio 3.34, 95% confidence interval 1.22–9.13, and *p* = 0.018).

Issues related to adherence to treatment were also common and were seen in 21.3% patients. Age and financial/employment status were significant independent predictors of nonadherence in multivariate logistic regression model (Tables 4(a) and 4(b)). Increasing age was associated with a significantly lower risk of nonadherence. Compared to aged pension, disability pension was associated with a significantly greater risk of nonadherence (odds ratio 3.11, 95% confidence interval 1.10 to 8.84, and *p* = 0.033). Compared to employed patients, unemployed patients were significantly more likely to have treatment nonadherence (odds ratio 4.19, 95% confidence interval, 1.46 to 12.01, and *p* = 0.008).

## 4. Discussion

This study sought to assess the psychosocial challenges faced by patients with ESKD in an Australian population. Among the patients referred to social work, the majority were >60 years of age, male, born in Australia, on HD, unemployed, and reliant on government assistance. The most common social work consults related to patients with difficulties with adjustment, treatment nonadherence, management of finances, and domestic assistance. We found that age, timing of referral (before versus after starting RRT), financial status, and employment status were independent predictors of adjustment issues. Age, financial status, and employment status also were independent predictors of treatment nonadherence.

As defined by Beder, adjustment to dialysis is described in relation to how the patient adapts to the multitude of stressors posed by the routine and restrictions of treatment. Social work intervention aims to stabilise the individual with a view towards maintenance of functionality and return to work after initiation of treatment. Early intervention, especially in “at-risk” patient groups, has been shown to significantly decrease the degree of psychosocial maladjustment in new-start dialysis patients [10]. Our current study suggests that patients dependent on government assistance are most at risk of maladjustment in the Australian setting. These patients may therefore also be most likely to benefit from social work intervention. The KHA-CARI guidelines on CKD management suggest the involvement of the social worker in early stages of CKD [12]. Of note the majority (>60%) of patients in this study were referred after commencement of dialysis, whereas a substantial number of patients with adjustment issue were referred prior to RRT. Maladjustment has been associated with loss of employment, which is not uncommon among dialysis patients [10, 13]. Earlier referral to social worker may therefore provide an opportunity to more effectively address adjustment concerns. Importantly, earlier referral may see more new-start dialysis patients maintaining or returning to employment. Treatment nonadherence in ESKD has been widely researched and remains a challenge for the care of these patients globally. Studies in dialysis patients have shown the association between decreased adherence and increased rates of depression, hospitalisation, morbidity, and overall mortality [14–17]. Rates of nonadherence, risk factors, clinical implications, and appropriate interventions have been variably described in the literature, largely owing to the heterogeneity of trials [18]. Treatment nonadherence was identified in 21.3% of patients in this study. We found that younger age, patients on disability pension, and unemployed patients were at a significantly higher independent risk of treatment nonadherence. This suggests that interventions directed at reducing disability and unemployment could improve treatment adherence.

Psychosocial interventions have proven to improve outcomes in randomised trials. Cukor et al. showed that cognitive behavioural therapy improved depressive symptoms, QoL, and treatment compliance in HD patients [18]. A systematic review by Chan et al. showed the association between psychosocial variables and QoL in dialysis patients concluding that targeted interventions to treat psychosocial factors may improve quality of life, morbidity, and ultimately mortality in this population [19]. This has implications for the findings of this study. Firstly the psychosocial factors identified in this population may represent surrogate markers of patient QoL; correlation of the findings with validated QoL scores would be of interest. Secondly, QoL scores may be an appropriate way to assess the outcome of social work interventions over time.

Limitations of our study were that data were collected retrospectively from a single tertiary centre social work database. The findings may therefore underrepresent the true extent and nature of psychosocial issues faced by ESKD patients. Efficacy of social work interventions was not measured, another limitation of our study. Also the number of encounters per patient was not tracked over the study's duration. This could have identified specific risk factors and/or groups that required more intensive social work follow-up.

In conclusion, this study observed the demographics of ESKD patients referred to social work at a tertiary hospital and found the major issues to be related to adjustment, financial difficulty, and domestic assistance. Risk factors for treatment nonadherence included age, disability pension, and unemployment. Adjustment issues were common and were more likely to be present in patients with younger age and referred before start of RRT. Patients with financial stability were less likely to have adjustment issues compared to patients on aged pension. Age was the common significant variable in adjustment and adherence to treatment. This highlights the need for further financial assistance/support from government and nongovernment agencies. Furthermore, we propose the need for earlier and more comprehensive social work support as patients approach ESKD which may lead to improvements in QoL, morbidity, and mortality.

## Figures and Tables

**Table 1:** Baseline characteristics of patients of CKD stage 5.

Characteristics	*N*	Mean (SD) or *n* (%)
Gender		
Male	244	148 (60.7%)
Age		62.4 (16.9)
Country of birth		
Australian		152 (62.3%)
Marital status		
Married		93 (38.1%)
Single		55 (22.5%)
Divorced		39 (16%)
Widowed		36 (14.8%)
Partner/Defacto		21 (8.6%)
Financial		
Aged pension		114 (46.3%)
Disability pension		86 (35%)
New-start allowance		12 (4.9%)
Parenting payment		2 (0.8%)
Salary		16 (6.5%)
Savings		13 (5.3%)
Youth allowance		1 (0.4%)
Employment status		
Employed		34%
Retired		77%
Unemployed		130%
Student		3%
Insurance		
Public		213 (87.3%)
Modality of treatment		
Transplant		32 (13.1%)
Haemodialysis		126 (51.6%)
Peritoneal dialysis		60 (24.6%)
None/conservative		26 (10.7%)
Time of referral		
Before RRT		94 (38.4%)

**Table 2:** Social issues present in patients with CKD stage 5.

Issue	*n* (%)
Adjustment	100 (41)
Finance	94 (38.5)
Domestic assistance	86 (35.2)
Transport	70 (28.7)
Caregiver stress	62 (25.4)
Nonadherence	52 (21.3)
Housing	45 (18.4)
Bereavement	41 (16.8)
Palliative care/advanced care planning	38 (15.4)
Mental health	36 (14.8)
Aged care	31 (12.7)
Employment	24 (9.8)
Child protection	6 (2.5)
Domestic violence	5 (2.0)

**(a):** 

Variables	Odds ratio	95% confidence interval		*p* value
		Lower	Upper	
Age	0.949	0.916	0.984	0.004
Pre-RRT issues	18.216	8.398	39.514	<0.001
Financial status				0.019
0: aged pension	Ref			Ref
1: disability pension	0.922	0.343	2.476	0.872
2: new-start allowance	0.555	0.086	3.569	0.535
3: parenting payment	0.181	0.002	19.751	0.475
4: salary	0.066	0.011	0.390	0.003
5: savings	0.100	0.16	0.647	0.016
6: youth allowance	5.147	0.000		1.000

**(b):** 

Variables	Odds ratio	95% confidence interval		*p* value
		Lower	Upper	
Age	0.960	0.933	0.988	0.005
Pre-RRT Issues	14.499	7.139	29.444	<0.001
Employment status				0.048
0: employed	Ref			Ref
1: retired	1.837	0.551	6.120	0.322
2: unemployed	3.344	1.225	9.128	0.018
3: student	0.444	0.018	10.746	0.618

**(a):** 

Variables	Odds ratio	95% confidence interval		*p* value
		Lower	Upper	
Age	0.968	0.938	0.999	0.041
Financial status				0.052
0: aged pension	Ref			Ref
1: disability pension	3.112	1.096	8.839	0.033
2: new-start allowance	0.588	0.072	4.790	0.619
3: parenting payment	4.109	0.000	—	0.999
4: salary	0.996	0.190	5.206	0.996
5: savings	0.766	0.113	5.187	0.785
6: youth allowance	0.000	0.000	—	1.000

**(b):** 

Variables	Odds ratio	95% confidence interval		*p* value
		Lower	Upper	
Age	0.953	0.930	0.977	<0.001
Employment status				0.008
0: employed	Ref			Ref
1: retired	1.256	0.289	5.461	0.761
2: unemployed	4.191	1.462	12.012	0.008
3: student	0.000	0.000	—	0.999
